# Virtual vs. real: exploring perceptual, cognitive and affective dimensions in design product experiences

**DOI:** 10.1186/s40359-023-01497-5

**Published:** 2024-01-02

**Authors:** Marta Pizzolante, Sabrina Bartolotta, Eleonora Diletta Sarcinella, Alice Chirico, Andrea Gaggioli

**Affiliations:** 1 Research Center in Communication Psychology (PsiCom), Universitá Cattolica del Sacro Cuore, Milan, Italy; 2 Department of Psychology, Università Cattolica del Sacro Cuore, Milan, Italy; 3 Applied Technology for Neuro-Psychology Lab, I.R.C.C.S, Istituto Auxologico Italiano, Milan, Italy

**Keywords:** User experience, Virtual reality, Virtual prototyping, Aesthetics, Emotions, Presence, Immersion

## Abstract

**Background:**

Virtual Reality (VR) has already emerged as an effective instrument for simulating realistic interactions, across various domains. In the field of User Experience (UX), VR has been used to create prototypes of real-world products. Here, the question is to what extent the users’ experience of a virtual prototype can be equivalent to that of its real counterpart (the real product). This issue particularly concerns the perceptual, cognitive and affective dimensions of users’ experiences.

**Methods:**

This exploratory study aims to address this issue by comparing the users’ experience of a well-known product, i.e., the *Graziella* bicycle, presented either in *Sumerian* or *Sansar VR* platform, or in a physical setting. Participants’ Emotional Engagement, Sense of Presence, Immersion, and Perceived Product Quality were evaluated after being exposed to the product in all conditions (i.e., *Sumerian*, *Sansar* and Physical).

**Results:**

The findings indicated significantly higher levels of Engagement and Positive Affect in the virtual experiences when compared to their real-world counterparts. Additionally, the sole notable distinction among the VR platforms was observed in terms of Realism.

**Conclusions:**

This study suggests the feasibility and potential of immersive VR environments as UX evaluation tools and underscores their effectiveness in replicating genuine real-world experiences.

## Background

VR offers promising advantages for simulating and assessing the dynamics of real-world interactions. VR technology allows individuals to be immersed in lifelike scenarios, situations, and contexts that closely resemble the corresponding real ones. However, VR also offers the possibility of simulating impossible worlds or unusual situations, promoting novel and unique experiences – i.e., liminal experiences [1, 2] self-transcendent [3] and out-of-the-body experiences [4, 5] which can lead to an altered perception of the self or to heightened affective and cognitive responses.

In recent times, thanks to the rapid widespread adoption of immersive technologies and software development, VR has gained popularity in the domain of User Experience (UX) for both research and design purposes [6, 7].

Firstly, VR allows researchers to create ecological immersive environments in which they assess individuals’ reactions and interactions towards products, and/ or services [8–11]. This, in turn, leads researchers to evaluate the final user while interacting with a product/service in its natural usage context, which is something not always possible in real life. Additionally, VR technology allows the collection of multiple information in the same session (e.g., psychophysiological measures, movement tracking, self-report measures, etc.), which, in turn, can lead to a deeper understanding of the individual’s perception, cognition and affect in response to the product/ service.

Secondly, UX designers can use VR to create and modify three-dimensional (3D) digital versions of physical prototypes, that is, Virtual Prototyping (VP) [12–15].

For example, using VR, designers can simulate a prototype of a car, including its exterior and interior. They replicate user interactions with controls, testing different environmental factors like road surfaces and weather conditions to assess their impact on the vehicle’s performance, safety, and overall user experience.

Therefore, VP brings numerous advantages by enabling real-time changes to 3D virtual models based on user feedback. This empowers architects and designers to make informed design decisions that prioritize user participation and functionality reducing time-to-market, cost savings, and knowledge sharing [16].

VR is an exceptionally versatile instrument for evoking unique characteristics that can significantly impact the user’s experience during interactions with the service or product. Various concepts are associated with virtual experiences, encompassing both device characteristics and the psychological states that arise from taking part in these experiences. For the purposes of this study, we will specifically concentrate on and, consequently, differentiate between the concepts of immersion and sense of presence.

According to the conceptualization introduced by Slater and Wilbur in 1997 [17], *immersion* is fundamentally a perceptual phenomenon. It relies on the objective technological capabilities of the device to provide a varied array of multisensory stimulation and tracking that maintain fidelity to real-world sensory modalities. The greater the fidelity achieved, the more the experience can be described as “immersive” [17]. Today, immersive technologies can deliver multisensory immersive experiences, by stimulating all the exteroceptive senses [18].

Tied to the concept of immersion, VR has been found to sustain the *sense of presence*, defined as the psychological sensation of really being within the virtual environment [19]. The sense of presence stands out as a key outcome in virtual experiences and has been employed in scholarly works as a metric for assessing the user experience [20]. It has been shown that the greater the sense of presence, the higher the capability to locate the self in the environment or situation, based on the perceived possibility to act in it [21, 22]. Research shows that sense of presence is also influenced by many factors including sensor fidelity, high-quality graphics, interactivity, and social presence [23–26].

Finally, VR has shown efficacy in eliciting emotionally resonant experiences - intense emotional responses, closely mirroring [27–29] or intensifying the feelings typically associated with real-world stimuli or events [30, 31] - evoking both simple emotions such as fear and joy [32] and complex emotions such as awe and interest, [33, 34] which, in turn, heighten the user’s sense of presence and immersion [35, 36].

Only a few studies have been conducted to compare UX of the same product/service in virtual vs. real settings [37–40]. Nevertheless, some of these studies have tangentially investigated how these features – namely immersion, sense of presence and emotional responses – differ between virtual and real products’ evaluations. This incidental examination emerged not from a direct intent to probe the depths of virtual versus real subjective experiences, but rather as a secondary consideration within research primarily centered on human-product interaction (i.e., ergonomics aspects, usability). Furthermore, these investigations have led to inconclusive results.

For instance, Kuliga et al. [38] reported similar levels of spatial presence and emotional engagement when comparing a virtual building to a real one. A study by Westerdahl et al. [37] showed that the virtual representation of an architectural model of a building was appreciated by participants since it gave veridical information about how the real building was perceived. However, users also acknowledged that the virtual model lacked sensory qualities and did not fulfill all the conventional criteria for providing a high degree of presence.

Furthermore, to our best knowledge, only one study [41] examined the relationships between presence, usability and user experience in a navigation task on a mobile app performed in a Cave Automatic Virtual Environment and in real life. The results showed a positive connection between the virtual field environment and hedonic qualities of the mobile app and confirmed the effect of usability on perceived presence [41].

To maximize the potential of VR as a valuable tool for UX research and design, it is essential to conduct more thorough studies that specifically explore the perceptual, cognitive, and emotional differences between virtual and real product interactions. In response to this need, the current exploratory study aimed to compare participants’ experiences—evaluating perceptual, cognitive, and emotional responses—when observing a design product, namely the Graziella bicycle, in virtual versus physical settings. This involved examining immersion, sense of presence and emotional engagement to understand how virtual experiences differ from their physical counterparts.

## Methods

**Graph 1:**
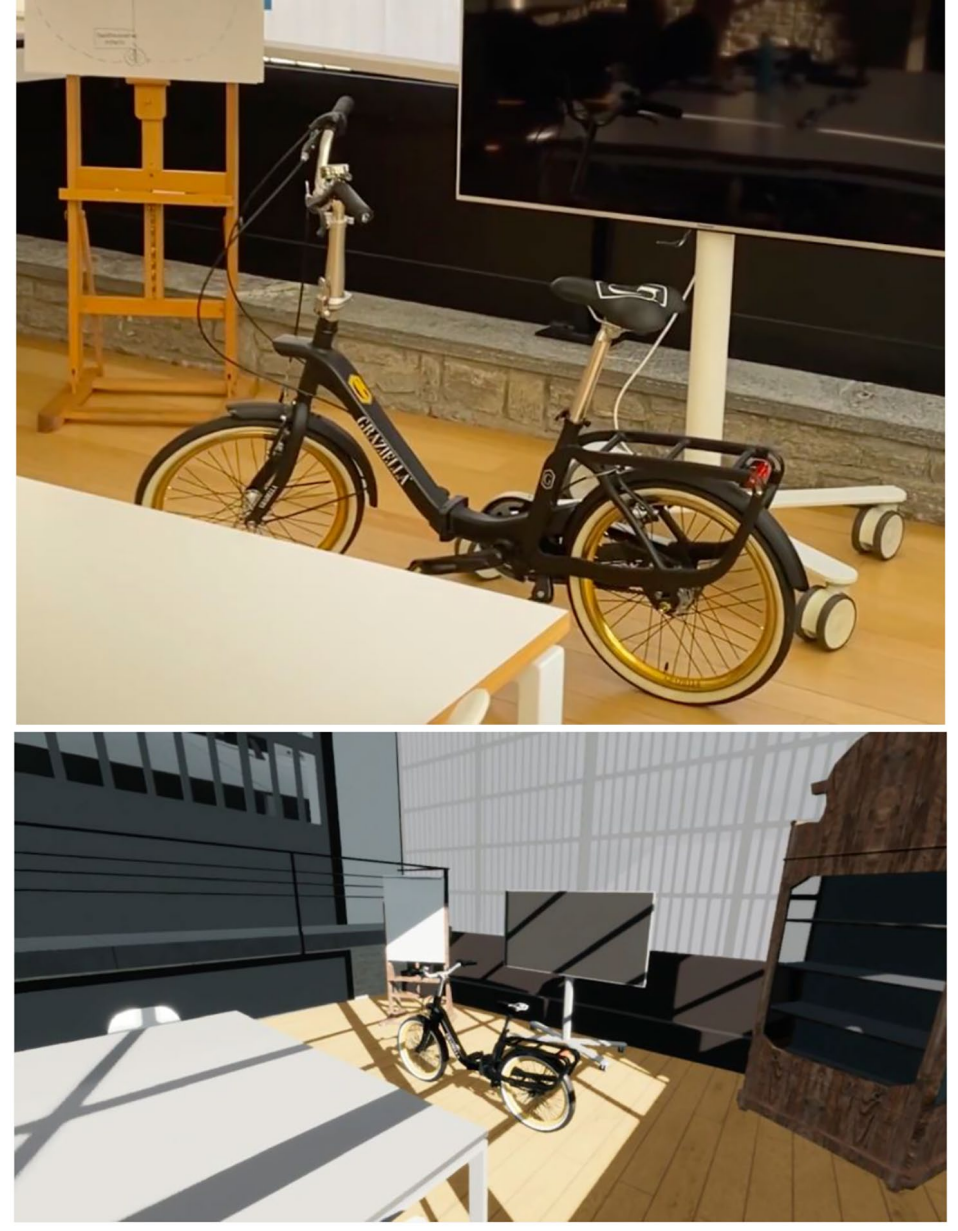
Above the *Sumerian* virtual environment; below the *Sansar* virtual environment

This exploratory study employed two specific virtual platforms: *Sumerian*, designed by *Amazon*^1^ and *Sansar*, developed by *Linden Lab.*^2^ (Graph 1)

The selection of the two platforms was informed by several reasons.

Compared to other game engine apps (e.g., *Unity, Unreal Engine*), both platforms are designed to be more accessible to non-developers (i.e., designers), allowing users to create VR experiences without extensive programming skills. However, they also present distinct features.

*Sumerian*, an *Amazon Web Services (AWS) product*, is recognized for its ease of use and rapid implementation. Thus, it enables quick prototyping and iterative design, important aspects for UX studies. *Sansar*, developed by *Linden Lab*, offers higher advanced graphics and dynamic lighting effects. Its enhanced visual capabilities, including detailed texture rendering, make it an optimal choice for studies focusing on the perceptual aspects of virtual experiences.

By evaluating the same product in both environments, we aimed to discern how these platforms differently influence users’ perceptual, cognitive, and emotional experiences. The unique attributes of each platform - *Sumerian*’s user-friendly design and *Sansar*’s focus on high-fidelity graphics - provided a comprehensive framework for understanding the potential of VR in UX design.

Consequently, the specific goals of the current study are as follows:

1. To compare the “real” and “virtual” experiences of the same design product by investigating several dimensions including immersion, sense of presence, emotional engagement, and perceived quality of the product.
2. To compare the two virtual experiences by means of *Sumerian* and *Sansar* platforms while evaluating the same dimensions mentioned above. The primary aim of this analysis is to identify the main strengths and weaknesses of each platform within a potential design application context.

Ultimately, this exploratory study aims to assess whether immersive virtual product presentation can function as an efficacious alternative or supplementary approach to physical product presentation in the realm of UX application.

### Participants

A total of 62 participants took part in the study (M_age_ = 36.10, SD_age_ = 21.12).

Participants were recruited through a convenience sampling method, primarily targeting individuals with prior experience in VR to ensure familiarity with the technology used in the study. This approach was chosen due to its efficiency and the ease of accessing participants within our network who met the study’s requirements.

Inclusion criteria required participants to be over 18 years old and to possess prior experience with VR. Also, exclusion criteria included history of severe motion sickness or vestibular disorders, uncontrolled epilepsy or seizures triggered by visual stimuli, pre-existing visual impairments that could be exacerbated by VR, claustrophobia or anxiety triggered by enclosed environments.

The participants were divided into three groups for the study conditions:

1. For the *Sumerian* platform (CSum), 21 subjects were recruited, comprising 14 females and 7 males (Mage = 44.05, SDage = 21.12).
2. For the *Sansar* platform (CSans), 21 subjects were recruited, comprising 11 females and 10 males (Mage = 39.29, SDage = 17.10).
3. For the physical presentation of the bicycle (CPhys), 20 subjects were recruited, of which 8 were females and 12 males (Mage = 36.10, SDage = 15.84).

### Tools

As described before, the design product, the *Graziella* bicycle, was presented either to participants as a virtual version on the *Sumerian* (CSum) or the *Sansar* (CSans) VR platform, or in physical presence (CPhys) in the main meeting room of a design studio in Italy. Participants belonging to the virtual conditions observed the virtual version of the product through an *HTC Vive Pro* Head Mounted Display (HMD), which included wireless controllers and two sensors for tracking and mapping users’ movements in the virtual environment.

### Procedure

A *between-subjects* study design was employed with participants randomly assigned to one of the three conditions. All participants signed an informed consent prior to the session, ensuring anonymity through ID codes, and completed a pre-exposure questionnaire.

For virtual product experience conditions (CSum, CSans), participants were informed that they would be immersed in a virtual environment simulating the main meeting room of the design studio. They could move using wireless controllers and were instructed to observe the bicycle, avoiding interaction, and focusing solely on its visual qualities.

For the physical presentation of the product (CPhys), participants could freely move inside the main meeting room of the studio to observe the *Graziella* bicycle, without interacting with it.

Also in this condition, participants were invited to focus on the visual characteristics of the product. The duration of each session was predetermined and fixed at approximately 10 min for all conditions. This timeframe was selected to balance adequate participant engagement with the product while minimizing potential fatigue, especially in the VR conditions. At the end of this fixed period, the experimenter indicated that the session had concluded and asked the participant to remove the HMD and to complete the post-exposure questionnaire.

### Measures

Before the experimental session, participants completed a pre-exposure questionnaire which included demographic information such as gender, age, qualifications, place of residence, occupation, previous VR experiences.

At the conclusion of the experimental session all participants in each condition filled out a post exposure questionnaire which included three different scales for CSum and CSans and all the scales apart from the *Objects Presence Questionnaire* (OPQ) for CPhys.

- The *ITC - Sense of Presence Inventory* (ITC-SOPI), [42] in its Italian adaptation, evaluated participants’ degree of immersion and presence and engagement within the virtual or the physical environment. It measures four different dimensions: *Sense of Physical Space, Engagement, Ecological Validity* and *Negative Effects*. Lessiter et al. [42] defined each group as such:

- *Sense of Physical Space*: sense of physical space in environment, interaction with and control over the parts of the environment;
- *Engagement*: sense of being psychologically immersed and enjoying the content presented;
- *Ecological Validity*: sense that the environment is lifelike real and resemble a real context/situation;
- *Negative Effects*: sense of adverse physiological reactions to the environment presented. Participants rated each item on a 5-steps Likert scale (from 1 = totally disagree to 5 = totally agree). This subscale aims at identifying adverse physiological reactions that participants might experience with immersive technology, such as cybersickness.

Items in some of these subscales (i.e., *Sense of Physical Space* and *Ecological Validity*) were adapted to specifically refer to the physical environment in which the experience occurred, following guidelines by Usoh et al. [43] and Nisenfeld [44].

- The *Objects Presence Questionnaire* (OPQ), [45] in its Italian adaptation, was used to capture the extent to which participants felt engaged with the elements within the simulated environment. Given the aim of this scale, results of the OPQ have been analyzed only between CSum and CSans conditions. The OPQ includes three different subscales: the *Involvement*, the *Realism* and the *Quality of Interface* (QoI) subscales.

- The *Quality of Interface* dimension measures the perception of product quality and usability. It indicates how positively the user assesses the ease of use, clarity, and effectiveness of the product;
- The *Realism* dimension assesses how much an individual perceives the product realistic in its usage context;
- The *Involvement* dimension measures how much participants are absorbed by the experience and responsiveness of the application.

- The 20-item *Positive and Negative Affect Schedule* (PANAS) questionnaire, [46] in its Italian validated version [47] was used to evaluate the emotional state of the participants at the baseline and after each experience. The scale comprises a *Positive Affect* (PA) subscale, assessing positive feelings or emotions such as joy, enthusiasm, satisfaction, and energy and a *Negative Affect* (NA) one evaluating negative feelings or emotions such as sadness, anxiety, anger, and fear.

## Results

Data analyses were performed through the statistical software IBM SPSS 23.0 and R programming language (version 4.2.3) with the ggplot2 package implemented for data visualization.

### Preliminary check

An inspection of Kurtosis and Skewness was conducted to determine if variables were normally distributed. All variables emerged as normally distributed. As a preliminary check, a series of one-way analysis of variance (ANOVAs) were conducted to check for possible participants’ differences at baseline in the PANAS and in the previous experience with VR measures between conditions. These analyses revealed no significant differences across conditions in terms of these pre-exposure variables.

### Self-report measures analyses

One-way ANOVAs were conducted to compare results in the ITC-SOPI and PANAS in the three different conditions (Table 1):

**Table 1:** Results of One-way ANOVAs for the ITC - SOPI and PANAS measures.

	Condition	Mean	SD	One-way ANOVA (Equal variances assumed)		
				F (df = 2)	*P*-value	Eta squared (η2)
*Sense of Physical Space*	CSum	67.05	13.39	1.57	0.216	0.051
	CSans	66.62	12.78			
	CPhys	59.50	18.97			
*Engagement*	CSum	52.24	9.09	19.20	.**001**	0.394
	CSans	52.86	7.61			
	CPhys	38.50	8.27			
*Ecological Validity*	CSum	19.62	4.22	0.974	0.383	0.032
	CSans	19.14	4.49			
	CPhys	21.00	4.50			
*Negative Effects*	CSum	8.81	6.80	2.08	0.134	0.066
	CSans	9.57	4.15			
	CPhys	6.65	1.66			
*Positive Affect*	CSum	38.10	8.40	5.16	.**009**	0.149
	CSans	39.81	5.76			
	CPhys	32.95	6.83			
*Negative Affect*	CSum	13.67	11.32	0.852	0.432	0.028
	CSans	11.00	1.76			
	CPhys	11.65	3.83			

The results showed significant differences in the *Engagement* subscale scores, measured through ITC -SOPI (F = 19.203, p = .000, η2 = 0.394). Subsequent post-hoc pairwise comparisons, corrected with *Bonferroni* test, specifically showed that both CSum and CSans significantly differ in terms of *Engagement* with respect to CPhys (p = .00; p = .00). No significant differences were observed between CSum and CSans (p = .969).

**Graph 2:**
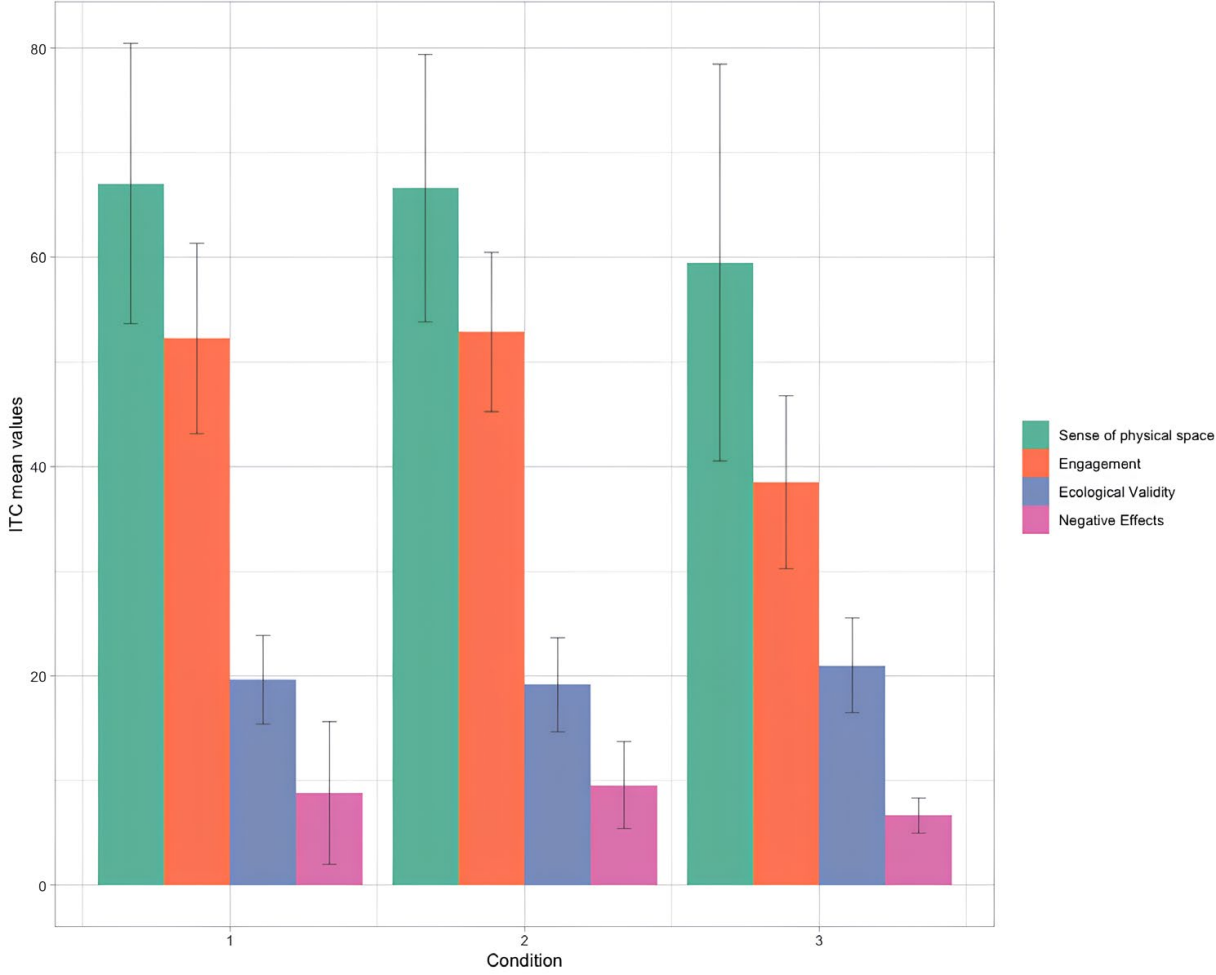
Bar plot depicting mean values for the ITC-SOPI measure for CSum (1), CSans (2) and CPhys (3).

Specifically, CSum (M = 52.24, SD = 9.09) and CSans (M = 52.86, SD = 7.61) obtained significantly higher scores than CPhys (M = 38.50, SD = 8.27) (Table 1; Graph 2).

The results showed significant differences also in the *PA* subscale scores, measured through the PANAS (F = 5.162, p = .009, η2 = 0.149).

Subsequent post-hoc pairwise comparisons, corrected with *Bonferroni* test, specifically showed that CSans showed a significant difference in *PA* subscale compared to CPhys (p = .008). No significant differences were observed between CSum and CPhys (p = .060) and between CSans and CSum (p = .715).

**Graph 3:**
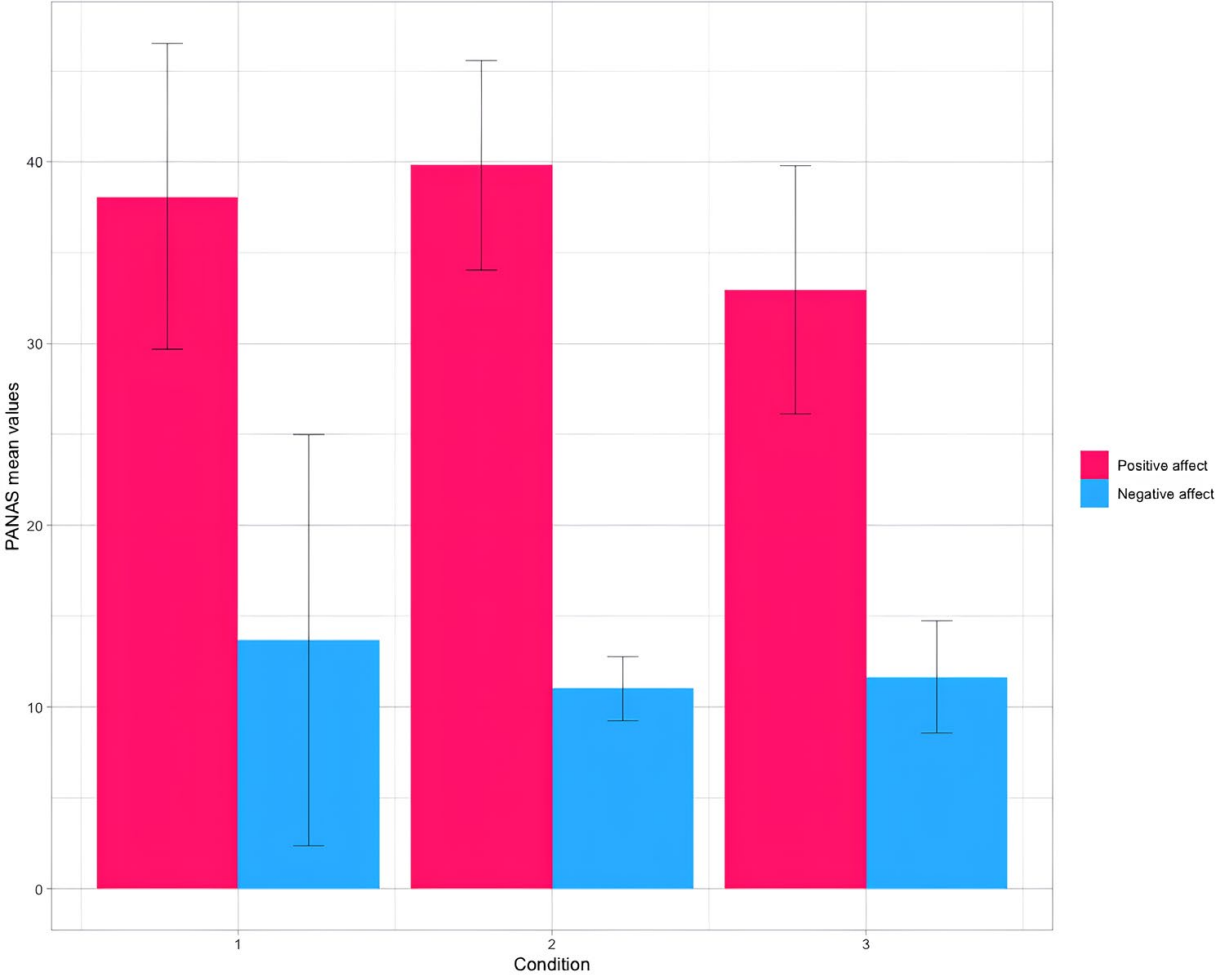
Bar plot depicting mean values for the PANAS measure for CSum (1), CSans (2) and CPhys (3).

Specifically, CSans obtained a significantly higher score (M = 39.81, SD = 5.76) than CPhys (M = 32.95, SD = 6.83) (Table 1; Graph 3).

Independent samples t-tests were conducted to compare results in the OPQ between the two virtual conditions (i.e., CSum and CSans).

The results showed a significant difference in the *Realism* subscale scores (t = − 0.127, p = .027, df = 40). Specifically, CSum (M = 40.95, SD = 4.97) obtained significantly higher scores than CSans (M = 41.19; SD = 6.99) (Table 2).

**Table 2:** Results of Independent samples t-tests for the OPQ measure

	Condition	Mean	SD	Independent samples t-tests	
				t (df = 40)	*P*-value
*Quality of Interface*	CSum	8.71	4.45	0.365	0.974
	CSans	8.24	3.99		
*Realism*	CSum	40.95	4.97	− 0.127	**0.027**
	CSans	41.19	6.99		
*Involvement*	CSum	17.71	3.22	0.252	0.901
	CSans	17.48	2.89		

Furthermore, we separately calculated correlations among *PA* and *NA* measures and the ITC-SOPI, for each experimental condition, in order to assess potential relationships between affective states and participants’ degree of immersion/ presence and engagement within the environment (Table 3).

Regarding CSum, PA positively correlated with the ITC SOPI subscales *Sense of Physical Space* (r = .812, p = < 0.001), *Engagement* (r = .810, p = < 0.001) and *Ecological Validity* (r = .606, p = < 0.001). Moreover, for CSum PA also negatively correlated with the ITC SOPI subscale *Negative Effects* (r = − .585, p = .005). For CSum, NA negatively correlated with the ITC SOPI subscales *Sense of Physical Space* (r = − .689, p = < 0.001), *Engagement* (r = − .731, p = < 0.001) and *Ecological Validity* (r = − .569, p = .007), and positively correlated with *Negative Effects* (r = .972, p = < 0.001).

Regarding CSans, PA positively correlated with the ITC SOPI subscales *Sense of Physical Space* (r = .490, p = .024) *and Engagement* (r = .598, p = .004). For CSum, NA positively correlated with *Negative Effects* (r = .506, p = .506).

Regarding CPhys, PA positively correlated with the ITC SOPI subscales *Sense of Physical Space* (r = .447, p = .048) *and Engagement* (r = .726, p = < 0.001).

**Table 3:** Pearson correlations computed between ITC-SOPI and PANAS dimensions (NA and PA) among CSum, CSans and CPhys.

Pearson Correlations					
	Condition	*Positive Affect (PA)*	*P*-value	*Negative Affect (NA)*	*P*-value
*Sense of Physical Space*	CSum	0.812**	**< 0.001**	− 0.689**	**< 0.001**
	CSans	**0.490** ^*^	**0.024**	− 0.238	0.299
	CPhys	**0.447** ^*^	**0.048**	0.374	0.104
*Engagement*	CSum	**0.810** ^**^	**< 0.001**	− 0.731**	**< 0.001**
	CSans	**0.598** ^**^	**0.004**	0.011	0.962
	CPhys	**0.726** ^**^	**< 0.001**	0.265	0.259
*Ecological Validity*	CSum	0.606**	**0.004**	− 0.569**	**0.007**
	CSans	0.232	0.311	− 0.170	0.460
	CPhys	0.080	0.736	0.148	0.534
*Negative Effects*	CSum	− 0.585**	**0.005**	0.972**	**< 0.001**
	CSans	− 0.097	0.674	**0.506** ^*^	**0.019**
	CPhys	0.383	0.096	0.273	0.245

**. Correlation is significant at the 0.01 level (2-tailed)

*. Correlation is significant at the 0.05 level (2-tailed)

## Discussion

Previous research has highlighted a significant gap in our understanding of the perceptual, cognitive and affective aspects of experiencing a product in a virtual context compared to its physical counterpart.

The primary goal of this study was to address this gap by comparing the experience of a design product, the *Graziella* bicycle, in virtual vs. real environments. Specifically, this study aimed at assessing the Sense of Presence, Immersion, Emotional Engagement and the Perceived Quality of the Product within these settings. Concerning the virtual environments, the study compared the characteristics of two virtual platforms - *Sumerian and Sansar* for assessing potential differences among different types of platforms.

### Virtual vs. physical experience of the product

The results of our study showed differences in two dimensions between the virtual (CSum, CSans) and the physical experience of the product (CPhys); *Engagement* and *Positive Affect*.

Participants belonging to the virtual conditions reported greater levels of *Engagement* with respect to participants belonging to the physical one. We know that the novelty factor of VR typically contributes to increased engagement. Participants are likely to find virtual experiences intriguing and exciting [48, 49], as VR technologies still represent a relatively novel and cutting-edge phenomenon. However, given that our inclusion criteria included having some prior experience with VR technology, we could presumably exclude that this finding is solely related to the so-called “wow effect”, linked to the intrinsic capacity of immersive technologies (e.g., Virtual Reality, Augmented Reality, and online videos) to generate awe experiences [50].

Also, if we look at earlier conceptualizations of *Engagement*, within the framework of information-system development, predominantly considered it as a process [51], namely, as a task-oriented aspect. In this perspective, engagement is seen as a step-by-step sequence or set of activities within the broader development process. It emphasizes the actions and tasks undertaken during the creation or implementation of an information system, often focusing on the efficiency and effectiveness of these processes.

Contrarily to this, conceptualizing *Engagement* as a quality of the user experience places emphasis on the subjective and experiential aspects of users’ interactions with technology. In this view, engagement is not solely a procedural outcome but rather a nuanced and intrinsic attribute of how users perceive and interact with the technology [52]. It encompasses emotional, cognitive, and sensory dimensions, reflecting the depth and richness of the user experience with VR technology. In light of these considerations, we could hypothesize that the heightened engagement observed within the VR conditions may have introduced a positive bias in the overall evaluation of the experience. This potential bias necessitates a cautious interpretation of the higher engagement levels in VR compared to the real world. It is possible that the immersive and novel aspects of VR technology may amplify users’ perceived engagement, overshadowing certain limitations or less engaging aspects of the VR experience that might become more apparent with repeated or prolonged exposure. Therefore, while the initial enhanced engagement in VR is an important finding, its long-term sustainability and impact on user experience require further investigation to fully understand the dynamics of user engagement in VR settings.

It is possible that our findings related to greater *Positive Affect* found in participants belonging to the virtual conditions might as well be related to this. Enhanced positive emotions during VR experiences compared to real ones has been found in other domains, such as education [53], architecture [38] and exposure to virtual vs. real nature [54].

For what concerns the physical condition, the significant positive correlation between *Engagement* and positive emotions suggests that, even in the absence of VR technology, user engagement relates to emotional experiences.

This aligns with research emphasizing the role of physical product engagement in influencing users’ emotional responses [55].

In conclusion, the results highlighted crucial factors inherent in VR during a virtual prototyping process, emphasizing the potential feasibility of integrating VR into UX testing.

The findings emphasize how this technology can make a virtual product experience credible, authentic, and, in some cases, psychologically more impactful than encountering the same product in a physical environment. This phenomenon leads users to perceive events within the virtual context as genuine occurrences, like reality [56, 57].

### Comparison between the virtual product experiences: Sumerian vs. Sansar

Notably, our results revealed no significant differences in ITC-SOPI dimensions between the two VR platforms, suggesting that they might elicit similar levels of sensor fidelity, perception of physical space, psychological involvement, and adverse reactions. However, a significant difference emerged in the OPQ subscale related to *Realism*, wherein Sansar obtained higher scores.

This difference in realism ratings can be attributed to *Sansar*’s advanced graphical capabilities, creating a more visually sophisticated virtual environment compared to Sumerian. The heightened realism in *Sansar* could have influenced participants to perceive the virtual environment as more authentic, thus resulting in elevated realism ratings compared to the *Sumerian* platform. This observation underscores the impact of graphical fidelity on users’ perception of realism in virtual experiences.

Furthermore, our study showed significant correlations between various dimensions of the ITC-SOPI and participants’ emotional states within both virtual environments. In the CSum condition, the Sense of Physical Space, Engagement, and Ecological Validity subscales exhibited positive correlations with Positive Affect and negative correlations with Negative Affect. In *Sansar*, these correlations were evident specifically for the Sense of Physical Space and Engagement, and only positive correlations were observed.

Thus, participants who reported heightened engagement and positive spatial perceptions in the virtual environments also reported experiencing more positive emotions. Conversely, those reporting negative effects in these virtual environments tended to experience higher levels of negative emotions. These findings align with existing literature on the relationship between presence (including dimensions like spatial presence, engagement, and ecological validity) and emotional engagement within virtual environments [36, 51, 58]. The positive correlations observed for virtual experiences are in line with studies suggesting that a heightened sense of presence and engagement in virtual environments can enhance emotional responses [22, 58]. This can be partially attributed to the immersive nature of well-designed VR, where users often suspend their disbelief and become emotionally involved in the virtual experience [19].

If, on one hand Suspension of Disbelief is not a necessary, credible, or explanatorily powerful component of presence experiences in VR, [59] on the other hand, it is optimized by the physical, functional, and psychological fidelity of the simulation [60]. Especially in design-related contexts, a more realistic environment, able to achieve a higher fidelity with the actual environment which the VR is emulating, elicits a better experience from the users [61].

The positive association between engagement and positive emotions, as well as the link between negative effects and negative emotions, underscores the importance of designing VR experiences and using VR tools that promote user engagement while minimizing potential discomfort or negative side effects [27, 30]. Such considerations are critical when aiming to create emotionally compelling virtual experiences.

Conversely, differences in the evaluations of the two platforms can be attributed to the unique characteristics inherent to each platform. As observed, *Sansar*’s enhanced graphics and dynamic lighting effects played a pivotal role in creating a highly immersive experience, in contrast to the relatively simpler visual aesthetics of the *Sumerian* platform. This discrepancy in visual fidelity and aesthetics likely contributed to variations in participant evaluations of the two platforms.

## Conclusions

The results of our investigation have unveiled minimal differences between the virtual and real evaluation conditions, particularly concerning perception of realism and sense of presence. This finding underscores the feasibility and reliability of utilizing VR for UX research, in line with prior research, [37, 38] which suggests a lack of substantial disparities in terms of object realism and environment presence between the two settings, affirming the promising utility of VR.

Nevertheless, our exploration extended beyond technical considerations into the realm of emotional engagement. Notably, our study revealed that participants reported heightened levels of engagement and positive affect in the VR conditions with respect to the physical one. Among the virtual platforms used, *Sansar* received higher evaluations across various product aspects considered in this study. These findings suggest that VR not only demonstrates technical feasibility but also holds psychological significance, offering motivation and positive emotional experiences to users.

However, it is crucial to acknowledge several potential caveats. Firstly, the imbalance in participant groups, with respect to gender. While our current analyses did not specifically examine differences between males and females in their virtual versus real experiences with the same product, it is imperative for future research in VR user experience to prioritize a more equitable distribution across gender groups. This approach will undoubtedly enhance the comprehensiveness of our insights, contributing to a richer understanding of the technology’s impact across different demographic groups.

Secondly, the volume of questions participants was required to answer may have imposed a cognitive burden on participants, potentially influencing their level of interest and engagement, particularly at the end of the questionnaire. Future studies have to consider reducing the length of the questionnaire, selecting the most relevant items.

Thirdly, as already highlighted in the Discussion, the heightened engagement observed within the VR conditions may have introduced a positive bias in the overall evaluation of the experience. While participants reported more positive emotions in the virtual environment, this emotional uplift should be approached cautiously when interpreting the overall product assessment. Although our study represents an exploratory effort, it underscores the strategic and innovative potential of VR for evaluating UX concerning physical products. To further enhance the realism and effectiveness of virtual product UX, conducting a comprehensive evaluation replicating tactile experiences of materials and textures from the virtual product would be beneficial. Such an approach could not only improve visual impressions but also enhance the perception of essential product attributes. Future research trajectories might consider intensifying interaction with virtual products, engaging vital sensory channels like sight, hearing, and touch [62–64]. Additionally, incorporating other type of measures (i.e., physiological) [65] and examining the potential to gauge purchase intent, a fundamental aspect in UX research and design, could further enhance our understanding of user experiences with these virtual environments [11].

Furthermore, considering the evolving landscape of social virtual environments, notably the *Metaverse*, there exists the opportunity to enhance the evaluation of product UX in virtual settings. Simulating social contexts within these environments could provide a more ecologically valid representation of real-world product usage, offering a comprehensive and realistic backdrop for UX assessments.

## Data Availability

The datasets generated during and/or analyzed during the current study are available from the corresponding author on reasonable request.

## Footnotes

1 https://aws.amazon.com/it/sumerian/.

2 https://www.sansar.com/.
